# Assessment of Knowledge, Attitude, and Practices of Cardiovascular Medical Emergencies Among Dental Students: An Institutional-Based Cross-Sectional Study

**DOI:** 10.7759/cureus.48568

**Published:** 2023-11-09

**Authors:** Pillai Arun Gopinathan, Faisal Saleh Alammari, Sulaiman Abdulrahman Alsulaim, Fahad Ghazi Alotaibi, Abdullah Muqbil Alanazi, Abdalrahman Wasel AL Khammash, Mohammed Abdullah Alshehri

**Affiliations:** 1 Maxillofacial Surgery and Diagnostic Sciences, College of Dentistry, King Saud bin Abdulaziz University for Health Sciences, Riyadh, SAU; 2 Dentistry, College of Dentistry, King Saud bin Abdulaziz University for Health Sciences, King Abdullah International Medical Research Center, Ministry of National Guard Health Affairs, Riyadh, SAU

**Keywords:** knowledge, cardiac disease, medical emergencies, dental graduates, practice, attitude

## Abstract

Background: Dental health and treatment are impacted by cardiovascular disease trends, complications, and related treatments. Every dentist should be prepared to handle the most serious cardiovascular emergencies and should expect to encounter them. Colleges have liability toward students to receive sufficient instructions so that they are prepared and self-assured to handle potentially life-threatening circumstances. The aim of this study is to assess the knowledge, attitude, and practice (KAP) among dental students in the Kingdom of Saudi Arabia.

Materials and methods: A cross-sectional study was conducted from January to June 2023 on undergraduates, interns, and postgraduate students studying at the dental school. A pre-validated questionnaire with 22 close-end questions consisting of demographic data and KAP items related to cardiovascular medical emergencies was circulated through Google form links. Statistical analysis was conducted using the chi-square test and multinomial regression.

Results: Of the 252 participants, the students overall showed moderate to poor responses among the KAP items related to cardiac emergencies. Most of the students (85%) showed positive attitude toward basic life support (BLS) and 78% on automated external defibrillation (AED). The majority of students showed unsafe practice (86.1%) that is they have not faced a cardiac emergency in the dental clinic, while 63.1% don’t update their emergency skills. Postgraduate students compared to other groups showed superior responses in all three domains of KAP with statistical significance (p<0.000). The undergraduates showed moderate levels of knowledge and attitude response, but unsafe responses to practice items were observed.

Conclusion: This study depicts that dental students who will eventually become dental practitioners have a critical need for training in managing cardiovascular medical emergencies.

## Introduction

A medical emergency (ME) is an illness or acute injury that could put an immediate risk to patient's life [1]. It is usually triggered by longstanding dental procedures or by administration of local anesthesia [2,3]. Among the MEs, 90 % are considered mild, while 8% are very serious [3]. Due to advances in medicines and ongoing changes in social lifestyle, dentists are prone to face MEs at the dental office [4]. The frequency of MEs is almost 5.8 times to occur in a dental clinic rather than a medical clinic [4]. Among the reported MEs, the most common were vasovagal syncope, allergic reaction followed by angina pectoris, cardiac arrest, hypertensive crisis, epilepsy, and ingestion of foreign body [3,4].

Current literature evidence indicates that cardiovascular disease (CVD) and diabetes mellitus have a high incidence rate among the population in Saudi Arabia and are leading factors for MEs [1]. The European-based study reveals that “sudden cardiac arrest” affects around 3,50,000 to 700000 individuals annually [4,5]. The global mortality rate associated with CVD is around 17.3 million deaths per year [6]. Acute cardiovascular disorders like angina pectoris, myocardial infarction (MI), heart failure, and hypertensive crisis are common disorders in dental practice [7,8]. The co-existing CVD conditions and increased geriatric and medically compromised patients to the dentist are the most common reasons for hospital referrals [4]. Inadequate knowledge about management strategies could lead to either delay in treatment or legal complications [9]. Dental management of cardiac-related issues has been mentioned in the dental association websites, publications, and other relevant guidelines [10]. Every patient expects that a dentist needs to manage life-threatening situations and have a basic knowledge of emergency medical care as Goldberg quotes “when you prepare for an emergency, the emergency ceases to exist” [9].

Information on previous studies of MEs of dental students and clinicians has been assessed in Saudi Arabia [3,9] but is limited to the scope of cardiac issues related to MEs. Studies show that dentists all around the world still struggle to manage emergency circumstances in dental clinics, even after completing a recognized formal medical emergency training program from their undergraduate and graduate degrees [11]. Thus, exploring the emergencies associated with common cardiac conditions could help to identify the gaps and train the students at the grass root levels which would further improve the care and outcomes of the patient. Hence, this study was commenced with the aim of assessing the knowledge, attitude, and practice (KAP) of cardiovascular MEs among dental students. The objectives of the study were to evaluate the variation in KAP related to cardiovascular MEs based on age, gender, and different academic levels of study among dental students.

## Materials and methods

A cross-sectional study was conducted at the dental school in KSA from Jan 2023 to June 2023. The study received ethical clearance from the Institutional Review Board of King Abdullah International Medical Research Center with the protocol number SP22R/175/06. The dental students were asked to fill the closed-end, self-administered multiple-choice electronic questionnaires to measure the KAP-based questions on cardiac MEs. The questionnaires (google form link) along with the consent form were forwarded to respective students through email and WhatsApp. The questionnaire was in English language and the participants were informed about the questionnaire survey which was voluntary, and the responses of participants were kept confidential.

The inclusion criteria were dental students (Doctor of Dental Medicine) from different academic levels of D1, D2, D3, D4, interns, and postgraduates, while the exclusion criteria were students, interns, and postgraduates who refused to fill out the questionnaire. D1 students stand for dental students in the first year, D2 (dental students in the second year), D3 (dental students in the third year), and D4 (dental students in the fourth year), respectively. The nonprobability-based convenience sampling technique was used, while the sample size was calculated based on 5% margin of error, 95% confidence level, and estimated population response distribution of 50%. The sample size was calculated using the “Raosoft” sample calculating website (raosoft.com/samplesize.html). The estimated sample size was 377 participants.

The questionnaire consisted of four sections: demographic data (four questions), knowledge-based (12 questions), attitude-based (5 questions), and practice-based (five questions). For the knowledge and practice items, the correct answer to each question was scored as 1, responses of “do not know” or “no” didn’t receive any points. For attitude questions, the participants were asked to indicate their level of agreement on a 5-point Likert scale ranging from disagree strongly to agree strongly. Agree/agree strongly was scored as 1 and neutral/disagree/disagree strongly was scored as 0.

For the knowledge questionnaire, the minimum score was 0, and the maximum score was 12. Scores ≥9 were considered as good, 6-8 moderate, and ≤5 as poor. For attitude-based questionnaires if participant scores ≥3 was considered a positive attitude, while <3 was considered a negative attitude. In the practice-based questionnaires, respondents scoring ≥3 were assumed to practice safely & scoring <3 was unsafe. The validity of the questionnaire was assessed by the panel experts & subject specialty belonging to the institution to judge the relevance of the content. Content validity was performed according to Lynn (1986) [12]. The questionnaire was evaluated by six subject experts on a four-point ordinal scale. 1 = not relevant, 2 = somewhat relevant, 3 = quite relevant, and 4 = highly relevant. A scale of CVI 0.95 was obtained. Fifteen students were subjected to a pilot survey along with a questionnaire & these students were added to the final analysis [13]. Cronbach’s alpha was assessed for reliability after one week in the same students who were part of the sample, and the value was found to be 0.85.

Statistical analysis

The data was analyzed using IBM SPSS Statistics for Windows, Version 20 (Released 2011; IBM Corp., Armonk, New York, United States). The categorical values were expressed through numbers and percentages. Dependent and independent variables were assessed through the chi-square test and multinomial regression. The p-value (< 0.05) was considered statistically significant.

## Results

Baseline demographic data

**Figure 1:**
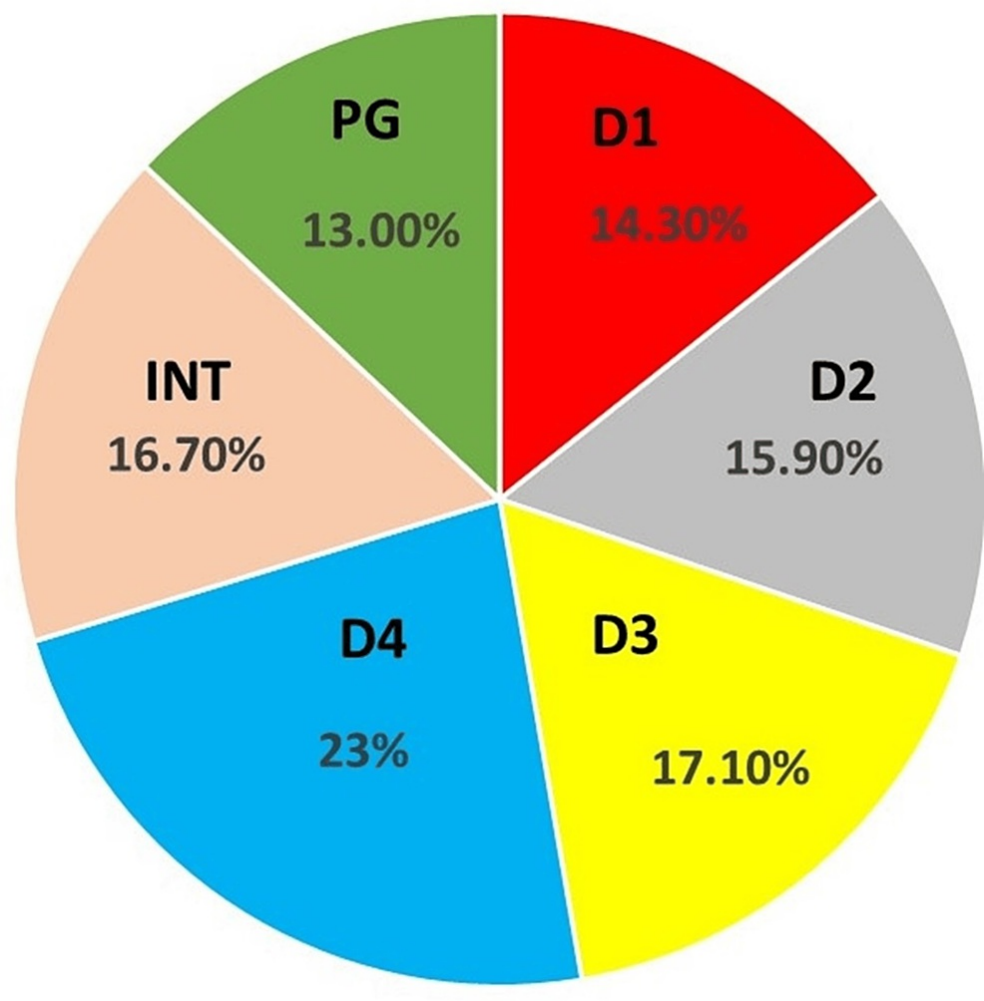
Percentage distribution of different academic levels of dental students D1: First-year dental students, D2: second-year dental students, D3: third-year dental students, D4: fourth-year dental students, Int: interns, PG: postgraduate students

A total of 377 dental students were eligible for study inclusion, of which 252 (66.84% response rate) consented to participate of which males were 134 (53.2%) and females were 118 (46.8%) in number. The average age of the students was between 20 and 26 years. The distribution of the sample population was based on different academic levels which were as follows: D1: 36, D2: 40, D3: 43, D4:58, interns: 42, and postgraduates 33 students, respectively (Figure 1). In the demographic characteristics, questions on exposure to informal-based cardiac-based education (independent variable) received a positive response of 63.1% (n=159). This question was tested for all the dependent variables of KAP and showed statistical significance with the chi-square test (p < 0.05).

Knowledge responses of students for cardiac MEs

**Figure 2:**
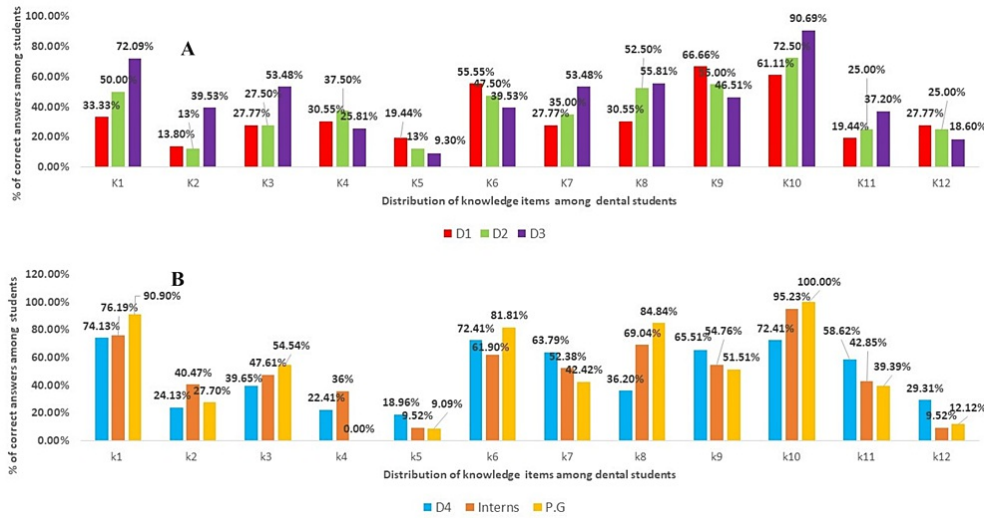
(A) Knowledge items among the first-year, second-year, and third-year dental students who responded with correct answers and (B) knowledge items among fourth-year, interns, and postgraduate dental students who responded with correct answers K1 to K12: Knowledge items from questions 1 to 12 D1: First-year dental students, D2: second-year dental students, D3: third-year dental students, D4: fourth-year dental students, PG: postgraduate students

The knowledge items were 12 in number, questions were based on epidemiology, risk factors, and warning signs based on cardiac medical emergencies (Table 1). Figures 2A, 2B depict the correct response of knowledge items among different academic dental students. The students showed good knowledge of questions on cardiac arrhythmia (81.3%) (n=204) and cause of chest pain (66.7% (n=168). Moderate knowledge was shown for questions on cardiac arrest (59.9%) (n=150), pedal edema (53.2%) (n=134), and myocardial infarction (57.1%) (n=143), while poor knowledge for the questions on the onset of acute MI (13.5%) (n=34) and metabolic equivalent of task (21%) (n=52) was observed (Table 1).

**Table 1:** Distribution of knowledge items with correct responses among different academic groups of dental students (p-value < 0.05 is statistically significant) K1 to K12: Knowledge items from questions 1 to 12

Sl. No	Knowledge-related questions	Correct answer	Percentage of correct response among different academic levels		Best academic group response to correct questions compared to other groups			Pearson Chi-square test analysis
			Percentage	‘n’	Group	‘n’	Percentage	
K1	What is the most common cause of chest pain?	Angina pectoris	66.7%	168	PG students	30	90%	.001
K2	Which among the following conditions has caused the most number of deaths with respect to cardiac disease?	Coronary heart disease	47.2%	118	Interns	17	40.47%	.001
K3	Cardinal Signs for Heart failure are	Dyspnea and fatigue	41.7%	105	PG students	18	54.54%	.022
K4	What is the most likely type of pain experienced in angina pectoris?	Dull /pressure type	25.8%	65	D2 students	15	37.50%	.006
K5	Onset of acute MI develops maximum during	Rest	13.5%	34	D1 students	7	19.44%	.009
K6	What is the most common cause of cardiac arrest in children?	Anoxia or airway obstruction	59.9%	150	PG students	27	81.81%	.001
K7	Why is aspirin given to a patient who has chest pain?	It prevents platelets from adhering together	47.6%	119	D4 students	37	63.79%	.221
K8	What could be the initial diagnosis if you had observed a distended neck veins and pedal edema during the case history scenario?	Congestive heart failure	53.2%	134	PG students	28	84.84%	.000
K9	The patient shows the signs of crushing falling in chest and experiences pain that radiates to neck and the mandible. what is your diagnosis?	Myocardial infarction	57.1%	143	D1 students	24	66.66%	.428
K10	What is cardiac arrhythmia ?	Abnormality in rate , regularity or site of origin of cardiac impulse	81.3%	204	PG students	33	100.00%	.000
K11	What is your preference for stress reduction protocol to ASA category 1 & II cases ?	Administer oral anxiolytic drugs	38.9%	98	D4 students	34	58.62%	.000
K12	What is Metabolic Equivalent of Task (METS) referred to as ?	Ability of patient to safely withstand stress related to dental treatment	21%	52	D4 students	17	29.31%	.001

When knowledge items were evaluated among different academic years of students, it was seen that postgraduate students answered most of the questions correctly and this was statistically significant (P< 0.05) (Table 1). This was followed by D4 students which also showed statistically significance. The least number of correct answers were given by D1 and D2 students which also showed statistical significance (p<0.009). The mean age group for knowledge items who answered the most questions correctly was from the age group of more than 26 years and showed statistical significance (p<0.002), while gender differences did not show any significance for the questions answered correctly.

Attitude responses of students for cardiac MEs

**Figure 3:**
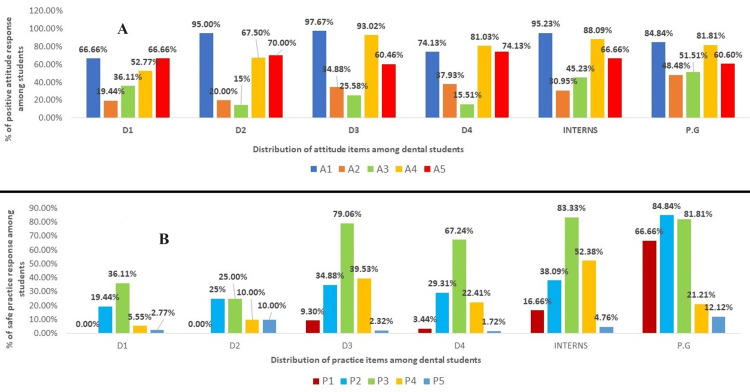
(A) Attitude items among different academic dental students who responded with positive attitude and (B) practice items among different academic dental students who responded to practice safely D1: First-year dental students, D2: second-year dental students, D3: third-year dental students, D4: fourth-year dental students, PG: postgraduate students A1 to A5: Attitude items from questions 1 to 5 P1 to P5: Practice items from questions 1 to 5

The attitude items were 5 five in number, and questions were based on management of cardiac emergency (Table 2). Figure 3A depicts a positive attitude response among different academic dental students. The majority of students showed positive attitude (85%) (n=214) toward certification of BLS; 78% (n=197) agreed the clinics should be equipped with the automated external defibrillation (AED). Figure 3B depicts safe practice response among different academic dental students. Among the student group, postgraduates and D3 students showed a significant statistical difference (p<0.000) for positive attitude toward basic life support (BLS) and AED. 67% (n=168) felt dental management of cardiac patients is difficult. Negative attitudes were seen toward cardio cerebral resuscitation (CCR) (67.9%) (n=171), while 70% (n=177) were not willing to perform mouth-to-mouth breathing. When attitude items were evaluated among the student group, postgraduates showed a significant statistical difference (p<0.000) for negative attitude toward mouth-to-mouth breathing (Table 2). The mean age group for attitude items who answered the most questions correctly were from the age group of more than 26 years and showed statistical significance (p<0.005), while gender differences did not show any significance for the attitude items.

**Table 2:** Distribution of attitude items with positive and negative attitude responses among different academic groups of dental students (p-value < 0.05 is statistically significant) A1 to A5: Attitude items from questions 1 to 5

Sl. No	Attitude-related questions	Agree/ agree strongly or positive attitude		Neutral/disagree/disagree strongly or negative attitude		Best academic group response to positive attitude compared to other groups			Pearson Chi-square test analysis
		percentage	‘n’	percentage	‘n’	Group	‘n’	percentage	
A1	Do you think dentist should be certified in basic life support courses (BLS)?	85.3%	214	14.7%	38	D3 students	42	97.67%	.000
A2	Give your opinion on the following statement: Cardio-cerebral resuscitation (CCR) is better than Cardiopulmonary resuscitation (CPR)?	32.1%	81	67.9%	171	PG students	16	48.48%	.063
A3	Will you be willing to perform a mouth-to-mouth breathing to a patient who has lost consciousness on the dental chair?	29.7%	75	70.3%	177	PG students	17	51.51%	.000
A4	Do you agree that the dental clinics should be equipped with automated external defibrillation (AEDs)?	78.2%	197	21.8%	55	D3 students	40	93.02%	.000
A5	Do you feel dental management in cardiac patients are difficult compared to other patients?	67%	168	33%	84	D4 students	43	74.13%	.708

Practice responses of students for cardiac MEs

The practice items were 5 in number, questions were based on alertness to avoid cardiac emergencies (Table 3). Most of the students (D1,D2,D3,D4) responded with unsafe practice that is 86%(n=216) did not face any cardiac emergency in the dental clinic, nor experienced a code blue situation (94%) (n=238) & 63.1% (n=159) do not update themselves with certified courses for emergency clinical skills (Table 3). When the practice items were evaluated among different academic-year students, post graduate students responded most to practice safely and it was statistically significant (p<0.000). Only the intern group responded to practice safety with respect to mock drills and was statistically significant (p<0.000). The mean age group for practice items who responded to practice safely was in the age group of more than 26 years and showed statistical significance (p<0.005), while gender differences did not show any significance for the attitude items.

**Table 3:** Distribution of practice items with safe and unsafe responses among different academic groups of dental students (p-value < 0.05 is statistically significant) P1 to P5: Practice items from questions 1 to 5

Sl no	Practice-related Questions	Yes or Practice safe		No/I don’t know or Practice unsafe		Best academic group response to practice safely compared to other groups			Pearson Chi-square test analysis
		percentage	‘n’	percentage	‘n’	Group	‘n’	percentage	
P1	Have you anytime faced a cardiac emergency at the dental clinic?	13.9%	36	86.1%	216	PG students	22	66.66%	.000
P2	Do you update emergency clinical skills by attending certified courses?	36.9%	93	63.1%	159	PG students	28	84.84%	.000
P3	If an emergency related to cardiac conditions occurs in the dental clinic, do you have access to nearby emergency facility?	62.7%	158	37.3%	94	PG students	27	81.81%	.000
P4	Have you any time performed a mock drill of management with respect to cardiac emergency in the clinics?	25.8%	66	74.2%	186	Interns	22	52.38%	.000
P5	Have you experienced a code blue situation?	5.2%	14	94.8%	238	PG students	4	12.12%	.172

Multinomial regression analysis

The multinomial regression analysis when evaluated revealed a significant model fitting of (0.000) and Nagelkerke R square value of 0.328 was observed (Table 4). When the dependent variable was analyzed with the independent variable (Table 5), it was observed that D2 dental students were more likely to agree (0.021) (p<0.05) with the need for a dentist to be certified with the BLS course. Further the students who had no exposure to informal cardiac-based emergency education felt significant (0.000) (p<0.05) need for dentist to be certified in BLS. There was no significant difference in agreement in gender with respect to the BLS course (Table 5).

**Table 4:** Model fitting information (p<0.05 is statistically significant)

Model	Model Fitting Criteria	Likelihood Ratio Tests		
	-2 Log Likelihood	Chi-Square	df	Sig.
Intercept Only	91.882			
Final	40.222	51.660	7	.000

**Table 5:** Dependent variable vs independent variable (parameter estimates) (p<0.05 is statistically significant) Higher value served as the comparison group

Do you think dentist should be certified in basic life support courses (BLS) ?		Significance	Exp(B)	95% Confidence Interval for Exp(B)	
				Lower Bound	Upper Bound
	Exposure to informal based cardiac based emergency education (NO)	.000	.116	.045	.302
	Exposure to informal based cardiac based emergency education (YES)	-	-	-	-
Agree/strongly agree	Academic year of study -D1	.857	1.130	.299	4.271
	Academic year of study -D2	.021	8.583	1.389	53.015
	Academic year of study -D3	.061	8.841	.902	86.642
	Academic year of study -D4	.383	.569	.160	2.019
	Academic year of study -Intern	.224	3.162	.494	20.247
	Academic year of study- PG	-	-	-	-
	Females	.530	.764	.330	1.768
	Males	-	-	-	-

## Discussion

MEs can occur with any individual, at any time or location, as well as during or after any dental procedure. Dentists need to understand that they are dealing with human life and should accept the dangers and obligations that come with the job as health professionals [14,15]. The present study results revealed that dental graduates and postgraduates are not competent enough to manage cardiac MEs, and they felt a need for more extensive training and have strong desire to acquire it. Numerous studies on MEs have been conducted on dental surgeons and dental students [5,16,17], but to our knowledge, very few or limited studies on cardiovascular issues have been addressed. Three patients who recently died in the dental office in Saudi Arabia were documented in the media as the result of poor clinic preparedness and failure of emergency management by the dentist [9]. Around 35% of the MEs are associated with systemic disease & cardiovascular disease [18]. The Brazilian Society of Cardiology estimates that 17.1 million individuals worldwide pass away each year due to heart disease or stroke [19].

In the present study, the overall performance of the students in the knowledge section who responded with the right answers was moderate level. The majority of students responded poorly to questions like signs for heart failure (41%) (n=105), pain experienced in angina pectoris (25.8%)(n=65), the onset of acute MI (13.5%) (n=34) and metabolic equivalent task (MET) (21%) (n=52). Similar findings were reported in numerous studies that acknowledged having little awareness of emergency situations, relevant medications, and equipment [5,20]. Also, dental graduates display a substantial knowledge gap about emergency circumstances, despite efforts made by medical institutions to provide the required information about managing many forms of MEs [21]. Only 21% (n=52) of the students could answer the question with respect to MET, which is a method of cardiovascular evaluation that has been verified and validated. To determine a patient's MET score, the functional physical activity of the patient is often examined through questionnaires or in-person interviews. The preoperative exercise capacity of patients scheduled for major surgical operations is estimated using the MET score [22]. Among the dental students, the majority of the postgraduate students, followed by interns and D4 students opted for the right answers. This could be due to higher academic degrees & prior training in cardiac patient treatment were all connected to better knowledge scores [10]. While low to moderate knowledge scores among D1,D2,D3 students could be due to lower academic levels, minimal clinical exposures to the management of cardiovascular patients, less training & not attending regular updated workshops [1,23].

Almost 85%(n=214) of students in the present study showed a positive attitude towards the BLS course. Studies have shown a relationship between experience and desire to learn and use the BLS procedure [4]. Saudi Arabia has made it mandatory in the curriculum for all health university programs to complete the BLS course before entering the clinics [21]. One of the studies revealed that very few dental practitioners have a comprehensive understanding of BLS [1]. Evidence, however, shows that dentists' BLS abilities degrade quickly. In a Polish study, 41.3% of dentists weren't adequately equipped to handle abrupt cardiac arrest [10]. However, several studies have revealed that over 50% of dentists worldwide are unable to successfully administer CPR [24]. Only 30% (n=75) in the present study agreed with mouth-to-mouth breathing, this can be attributed to respondents' worries about contracting airborne and other disease transmission [25]. One of the studies showed a similar percentage [26].

In the present study, 67.9%(n=171) of the students were unaware of the concept of Cardio cerebral resuscitation (CCR). This might be because of inadequate training, lack of infrastructure required or little exposure to CCR [27]. CCR is a substitute for CPR, as it is specifically used for out-of-hospital cardiac arrest situations. The main advantages of CCR are improved survival & cerebral function of the patient, eliminates mouth-mouth ventilation, prevents the use of endotracheal intubation during electrical and circulatory stages of cardiac arrest due to ventricular fibrillation [28]. The students who participated in the study (78%) (n=197) reflected with positive attitude towards the presence of AED in the dental clinic. Periodic hands-on instruction can boost users' confidence in their ability to use it. This is crucial for policymakers who need to implement the use of AED at the clinical level, thus to treat cardiac arrest more efficiently [29]. CPR in combination with early shock of a defibrillator is known to improve the outcomes of survival rate by up to 75% [25]. Even non-medical employees may be effectively trained to operate AED because they have been proven to be precise and efficient [30].

Overall, 67%(n=168) of students felt managing cardiac-based patients was difficult. Similar findings i.e. (50%) of the participants faced similar difficulty [10]. This could be due to numerous reasons, over the estimation of bleeding risk, the need for consultation from a physician before performing invasive procedures, the absence of a nearby cardiologist at the workplace, and need for antibiotic prophylaxis [10]. Students could familiarize themselves with the stress associated with life-threatening scenarios, by frequently visiting hospitals and emergency services. Dental students who participated in a four-week hospital-based program saw progress in the assessment and management of ME [24].

In the practice-related item, the majority of students (62.7%)(n=158) felt that they had access to a nearby medical facility in case of a cardiac emergency. This may be due to government dental clinics are typically situated near medical facilities, availability of emergency doctors for consultation along with advanced continued education courses updated on a regular basis [3]. Almost 74%(n=186) of students have not undergone mock drill management for a cardiac emergency, while 94%(n=238) have not faced a code blue situation and only 37% (n=93) have updated emergency skills. A study reported that only 12.9% of the private dental clinics performed periodic office mock drills [9]. Without practice, theoretical knowledge and demonstrations are insufficient to establish competency in managing medical crises [31]. Improvements in provider’s confidence, communication, teamwork, and patient safety are made when emergency situations are practiced in a monitored and simulated setting. It is a flexible strategy that promotes group cohesion in real circumstances [32]. According to the Resuscitation Council 2013, staff should receive refresher training on a yearly basis [31], while the European Resuscitation Council and American Heart Association advise dentists to update their knowledge of MEs every two years [11]. Numerous authors have emphasized the importance of receiving refresher training courses, particularly for people who don't regularly perform CPR [26].

In the present study among dental students, the postgraduate group compared to the undergraduate group revealed superior skills in KAP items, which were found to be statistically significant (p<0.000). This could be due to the level of education, training, and courses which are more extensive for post graduate programs [10]. D1,D2,D3 & Intern groups showed moderate to poor skills in all the KAP domains. Few studies have shown that dentists are underprepared when they graduate [14]. The mean age group for KAP items who answered the most questions correctly, who had a positive attitude & who practiced positively were from the age group of more than 26 years and showed statistical significance (p<0.002), (p<0.005), and (p<0.005) respectively. It was also observed that informal education on cardiac-based emergencies on the KAP items, in general, had a statistical significance (p < 0.05). This type of informal education could be received via family, friends, health care providers, internet, television & social media, or those who worked with cardiologists [10,33]. The gender differences didn’t show any statistical significance among any KAP items with respect to cardiac emergencies. A study conducted by Shaik et al. mentioned that gender has no significant impact on knowledge items [34].

The present study received a response rate of 67% (n=252) (estimated sample size of 377), which was comparable to cross-sectional research conducted in the UK using a questionnaire, where 74% of participants responded [3]. To guarantee the student full involvement in the study, the responders were contacted twice at their clinics and classes. Despite being good, the response rate was a little lower than anticipated. This is most likely a result of the students' hectic schedules at the clinics, semester exams, or lack of enthusiasm for taking part in the study.

Our study revealed a concerned picture in handling cardiac emergencies among dental students, and they expressed the need for further training. Considering that dental students have minimal knowledge of ME management and that there is less information regarding the value they place on being competent in this area of patient care. Although there are several studies done on MEs among dental students, there are significant differences in the undergraduate training offered around the globe. The students who become future dental practitioners undergo tremendous stress in managing cardiac patients & also have a low interest in this topic due to its complexity of medical literature.

Limitations of the study

The study was restricted to a single dental college and convenience sampling was used. There was no comparison between those who had BLS training and those who had received refresher training. Despite the limitations, our study provides insight, especially for the need of extensive cardiac training in the area specific to MET score, CCR, AED, updating emergency skills, and mock drill management for undergraduate students. Future research could be done with a larger and more heterogeneous sample in terms of combined dental schools and medical universities for better results.

## Conclusions

The study showed overall moderate to poor knowledge, neutral attitude, and unsafe practice of cardiac MEs among the undergraduate dental students. The postgraduate students performed better in the KAP domains of cardiac emergency. The training of practicing dentists must be enhanced at the undergraduate, graduate, and continuing education levels along with the setup of dental offices to handle such events. If the brain is tuned at the grassroot level, an emergency won't scare them, and they can handle the patient with assurance and effectiveness.
